# Immediate effect of lower extremity joint manipulation on a lower extremity somatosensory illusion: a randomized, controlled crossover clinical pilot study

**DOI:** 10.3389/fnhum.2022.1011997

**Published:** 2022-11-08

**Authors:** Shannon Schueren, Hugh Hunger, Huong Pham, Dean L. Smith, Charles Layne, Christopher A. Malaya

**Affiliations:** ^1^Research Center, Parker University, Dallas, TX, United States; ^2^Department of Kinesiology and Health, Miami University, Oxford, OH, United States; ^3^Department of Health and Human Performance, Center for Neuromotor and Biomechanics Research, University of Houston, Houston, TX, United States

**Keywords:** chiropractic manipulation, motor control, postural balance, somatosensory illusion, extremities

## Abstract

**Objective:** This study explored the influence of lower extremity manipulation on the postural after-effects of standing on an inclined surface.

**Methods:** Eight healthy individuals (28.0 ± 4.1 years) were recruited for this open-label, crossover study. Participants stood on an incline board for 3 min to develop a known form of somatosensory illusion. After randomization to either a lower-extremity joint manipulation or no intervention, participants immediately stood on a force plate for 3 min with eyes closed. After a 24-h washout period, participants completed the remaining condition. Center of pressure (CoP) position data was measured by a force plate and evaluated using statistical parametric mapping. Pathlength, mean velocity, and RMS were calculated for significant time periods and compared with corrected paired *t*-tests.

**Results:** Parametric maps revealed that CoP position of control and intervention conditions differed significantly for two time periods (70–86 s—control: 0.17 ± 1.86 cm/intervention: −1.36 ± 1.54 cm; 141–177 s—control: −0.35 ± 1.61 cm/intervention: −1.93 ± 1.48 cm). CoP pathlength was also significantly decreased for the second period (control: 6.11 ± 4.81 cm/intervention: 3.62 ± 1.92 cm).

**Conclusion:** These findings suggest that extremity manipulation may be a useful intervention for populations where CoP stability is an issue. This study contributes to the growing body of evidence that manipulation of the extremities can drive global postural changes, as well as influence standing behavior. Further, it suggests these global changes may be driven by alterations in central integration.

**Clinical Trial Registration:** ClinicalTrials.gov, NCT Number: NCT05226715.

## Introduction

Postural control has been defined as the act of maintaining, achieving, or restoring a state of balance during any posture or activity (Pollock et al., 2000). This requires an individual to regulate their body position and orientation, as well as respond to external perturbations (Maki and McIlroy, 1997; Shumway-Cook and Woollacott, 2017). Maintaining postural orientation is a complex task requiring the integration of the somatosensory, vestibular, and visual systems to orient the body with regards to the surface underfoot (Smart and Smith, 2001). A study by Marshall and Murphy (2010) found that altered paraspinal motor responses in participants with low back pain correlated with poor balance. Other studies have found alterations in kinesthetic sense (Heikka and Astrom, 1996) and standing balance (Sjostrom et al., 2003), as well as with pain following spinal injury (Brumagne et al., 2000; Descarreaux et al., 2005). These alterations have been shown to be influenced by decreases in joint position sense (Treleaven et al., 2003, 2006). Joint manipulation has previously been found to increase joint position sense (Gong, 2013) and maybe a valuable therapy in the treatment of kinesthetic balance disorders.

Previous investigations of spinal manipulation have found neurologically driven influences on the central nervous system including changes in cortical facilitation and sensorimotor integration, as well inhibition of motor responses (Haavik-Taylor and Murphy, 2007; Taylor and Murphy, 2008; Haavik et al., 2016). Changes in peripheral proprioception and muscle activations have also been reported (Gong, 2013; Kingett et al., 2019). Some researchers have posited that these changes are driven by an afferent barrage of peripheral somatosensory information affecting central integrative processing methods (Taylor and Murphy, 2008; Savva et al., 2014).

While there is mounting evidence for a central, neurologically active component of spinal manipulation, it is unclear if these effects are also present during the manipulation of joints of the extremities. Studies by Malaya et al. (2020), Malaya et al. (2021) have found task-based and postural changes from manipulation of the upper and lower extremities. The nature of these changes is not easily explainable as joint-localized phenomena—despite joint-specific application—and has been suggested to be centrally mediated, similar to the effects of spinal manipulation (Malaya et al., 2021). However, the relative contributions of central and peripheral mechanisms to the effects of extremity manipulation are still unknown.

Standing on a toes-up inclined surface has been seen to facilitate the development of a lasting somatosensory illusion; specifically, after stepping onto flat ground and closing their eyes, many individuals will adopt a forward lean proportional to the degree of incline experienced during the development of the illusion (Kluzik et al., 2005). This after-effect occurs regardless of the degree of incline, the direction of incline (anterior, posterior, lateral), static standing or stepping on an incline, and will still be seen in the forward lean of the trunk and head when the individual’s legs are blocked from movement (Kluzik et al., 2007a). Both the trunk and head lean seen with leg blocking in the post-incline period, as well as several EMG studies performed by Kluzik et al. (2007b) confirm that the lean adaptation seen in the post-incline period is not explainable by tonic peripheral musculature contraction; rather, the leaning appears to be a central postural adaptation that orients relative to the surface (Kluzik et al., 2007a; Young and Layne, 2020; Young et al., 2020).

This pilot study aimed to explore the influence of lower extremity manipulation on the postural after-effects of standing on an inclined surface. Previous studies support the idea that these after-effects (e.g., illusionary misperceptions) are of central nervous system origin (Kluzik et al., 2005, a, b; Wright, 2011). If manipulation of the extremity joints can modulate or influence a centrally mediated illusion, it is possible that joint manipulation also exerts an influence on the central nervous system. Understanding the mechanisms behind extremity-based joint manipulation and its potential effects on postural control could have broad implications for rehabilitation in clinical populations in which postural instability and falls are a health risk.

## Materials and Methods

### Participants

This study recruited a sample of eight healthy individuals (25% female) between the ages of 18 and 35 (28.0 ± 4.1 years) over a 3-week period prior to study date (see Table 1). Participants had no documented surgeries, neuromusculoskeletal injuries, or systemic diseases that could affect their ability to stand on an incline for 3 min with their eyes closed. Participants were not knowingly pregnant and weighed less than the force plate operating limit of 440 lbs. Written informed consent was obtained from each participant prior to the start of experimental procedures. Approval to conduct this study was granted by the Institutional Review Board at Parker University (A-00222), in accordance with the Declaration of Helsinki. No participants were lost to follow-up and all collected data were used in the analysis. There were no adverse events or unintended effects during the course of the study.

**Table 1:** Participant details.

**Participant ID**	**Age**	**Sex**	**Height**	**Weight**
	**(years)**		**(ft/in)**	**(lbs)**
1	34	Male	5’9”	158
2	29	Female	5’7”	150
3	24	Male	6’0”	165
4	32	Male	5’7”	160
5	29	Female	5’2”	107
6	24	Male	6’0”	200
7	34	Male	6’1”	195
8	26	Male	5’11”	170

Registered at ClinicalTrials.gov, NCT Number: NCT05226715.

### Study design

**Figure 1:**
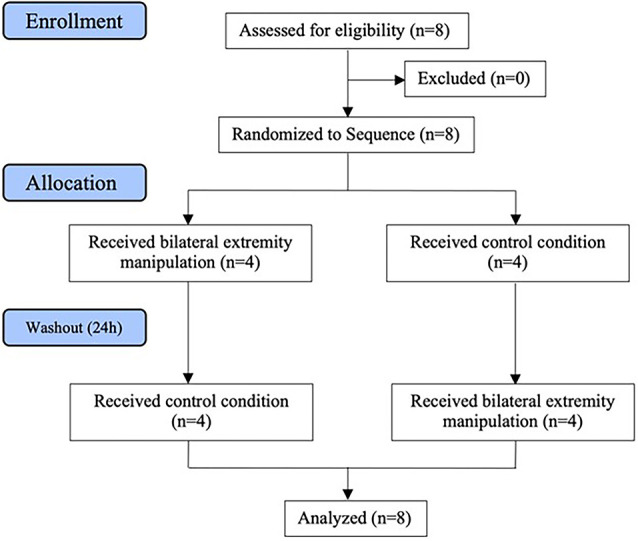
Consort diagram.

This study was an open-label, controlled, crossover clinical pilot. Participants were randomized into two different groups using block randomization *via* a randomized number generator by the treating practitioner. All other study coordinators were blinded to the intervention each participant received. Participants were asked to stand on a force plate with eyes closed for 3 min so that baseline center of pressure (CoP) could be obtained. Immediately following baseline CoP measurement Group 1 received a bilateral, lower-extremity manipulation series (described below) in the supine position (intervention) on the first day, and, after a 24-h washout period, they underwent all procedures leading up to the intervention, without the manipulation series itself taking place (control condition). Group 2 performed the control condition on the first day, and the intervention condition on the second day after the same 24-h washout period. The washout period was chosen in line with previous work by Malaya et al. (2020, 2021; see Figure 1).

CoP was assessed on a force plate after the development of the incline illusion and immediately after receiving either the intervention or control condition for all participants. All testing took place over two consecutive days at Parker University.

**Figure 2:**
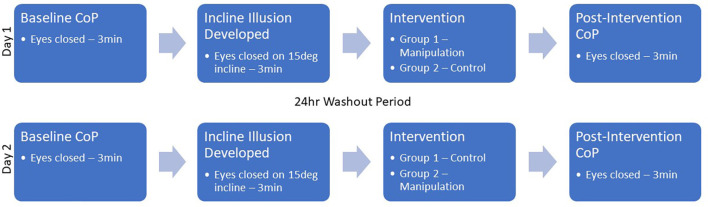
Study design.

The lower-extremity manipulation series consisted of a single high velocity, low amplitude manipulation bilaterally to the coxofemoral, tibiofemoral, and talotibial joints from proximal to distal in the supine position (as detailed in Malaya et al., 2020, 2021). Coxofemoral manipulation was performed in a long-lever superior to inferior thrust with bilateral web contacts on the distal thigh. Tibiofemoral manipulation was a long axis-distraction performed with an index contact on the distal tibia above the malleoli with a 5–10 degree knee angle. Talotibial manipulation was performed using a bilateral medial hand contact with thrust in the superior-to-inferior, posterior-to-anterior direction. All manipulations were performed bilaterally for each participant and by an experienced licensed chiropractor with expertise in extremity manipulation. The control condition required participants to lay supine on a chiropractic bench for 30 s without manipulation (see Figure 2). No adverse events were reported.

### Development of the incline illusion

Participants were asked to stand on a 15° inclined, toes-up ankle stretching board for 3 min with their eyes closed. Previous work on this topic has established that eyes-closed standing on an incline board for 2.5 min can facilitate the development of an illusory leaning after-effect during subsequent standing on flat ground with closed eyes (Kluzik et al., 2005, 2007b).

### Illusion response testing

Immediately after receiving the control or intervention (within 10 s), participants were asked to step off of the incline board and stand on a force plate for an additional 3 min. Participants were instructed to stand quietly, with their arms at their sides and eyes closed, and not to resist any tendency to lean in accordance with previous studies by Kluzik et al. (2005) which told participants to “not pay attention to your posture.”

### Data collection

Anterior to posterior CoP data were captured at 50 Hz with a BTracks^TM^ (Balance Tracking Systems) force plate using the Explore Balance software application (Balance Tracking Systems, version 2.0.4). All CoP data were low-pass filtered at 0.1 Hz with a 2nd order Butterworth filter to isolate slow postural changes and eliminate any fast fluctuations from stabilizing corrections (Gurfinkel et al., 1995; Fransson et al., 2002; Kluzik et al., 2005). Data were down-sampled in MATLAB using the resample function to achieve a total of 150 points, each representing a 1.2 s step in time (see Guthrie and Buchwald, 1991). CoP time series data from the control and intervention conditions were then compared directly using the statistical methods detailed below. CoP data were also used to calculate pathlength, mean velocity, and the root mean square of the lean behavior. In this study, pathlength is the cumulative distance traveled by each participant’s CoP on the force plate (Paillard and Noé, 2015). RMS is a measure of the magnitude of movement that each participant’s CoP varies with respect to the mean location (Paillard and Noé, 2015). All measures were calculated with a custom MATLAB script (MATLAB R2018b:9.5.0.944444).

### Data analysis

#### Power analysis

A *post hoc* power analysis using G*Power (v.3.1) for a sample size of eight participants yielded an effect size of 1.47 with a power of 0.94 at an α of 0.05.

### Primary outcome

**Figure 3:**
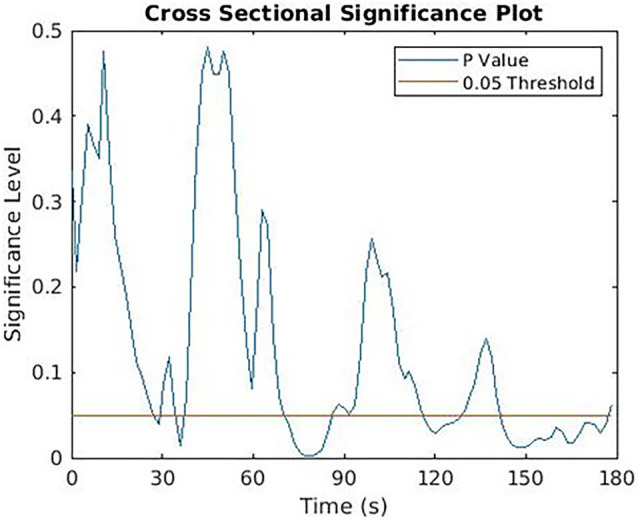
Cross sectional significance plot.

Differences between the control and intervention CoP time series were evaluated by creating a new series comprised of two-tailed *t*-tests for each time point shared between conditions (see Figure 3). Autocorrelation values of 0.915 were found for the data according to the methods outlined in Guthrie and Buchwald (1991). In line with previous studies utilizing this analysis, when the value of the *t*-series exceeded our set significance of 0.05 for at least 12 subsequent points (given a series of 150 points with autocorrelation of 0.915), the difference was considered significant (Guthrie and Buchwald, 1991; Urakawa et al., 2017, 2018). This analysis was conducted using a custom MATLAB script (MATLAB R2018b:9.5.0.944444).

### Secondary outcomes

Paired *t*-tests were used to compare pathlength, mean velocity, and RMS between the control and intervention conditions for significant time periods. Statistical significance was set at 0.05 and a Bonferroni correction for six pairwise comparisons was applied to account for any error from multiple comparisons (*p* < 0.05/6 = 0.0083). All calculations denote measures in the anteroposterior direction. All analyses were conducted using MATLAB (MATLAB R2018b:9.5.0.944444).

## Results

Overall, five separate time periods on the force plate contained values below our significance threshold of 0.05 (Figure 3). Of these original five, only two time periods had 12 or more continuous points below the significance level and could be considered statistically significant (Table 2). Pathlength, mean velocity, and RMS were calculated for the significant time periods and compared across conditions using Bonferroni corrected paired *t*-tests (Table 3).

**Table 2:** Significance and center of pressure (CoP) comparisons of time periods on force plate.

**Time range (s)**	**Consecutive points below**	**Control CoP mean ± Std (cm)**	**Intervention CoP mean ± Std (cm)**
27.6–30	2	0.52 ± 1.81	−0.09 ± 1.73
34.8–38.4	3	0.42 ± 1.75	−0.18 ± 1.27
70.8–86.4	13*	0.17 ± 1.86	−1.36 ± 1.54
116.4–129.6	11	−0.15 ± 1.86	−1.81 ± 1.79
141.6–177.6	30*	−0.35 ± 1.61	−1.93 ± 1.48

When the value of the *t*-series exceeded our set significance of 0.05 (denoted by *) for at least 12 subsequent points (given a series of 150 points with an autocorrelation of 0.9150), the difference was considered significant.

**Table 3:** Paired *t*-tests for pathlength, mean velocity, and RMS of significant time periods.

**Time range (s)**	**Pathlength mean ± Std (cm)**		**Mean velocity mean ± Std (cm/s)**		**RMS mean ± Std (cm)**	
	**Control**	**Intervention**	**Control**	**Intervention**	**Control**	**Intervention**
70.8–86.4	1.92 ± 1.37	1.94 ± 0.99	0.04 ± 0.19	0.01 ± 0.23	1.36 ± 1.34	1.67 ± 1.25
141.6–177.6	6.11 ± 4.81*	3.62 ± 1.92*	0.02 ± 0.28	0.00 ± 0.14	1.44 ± 0.85	2.14 ± 1.23

*Denotes a statistically significant difference between respective control and intervention conditions as determined by corrected paired *t*-test (*p* < 0.0083).

**Figure 4:**
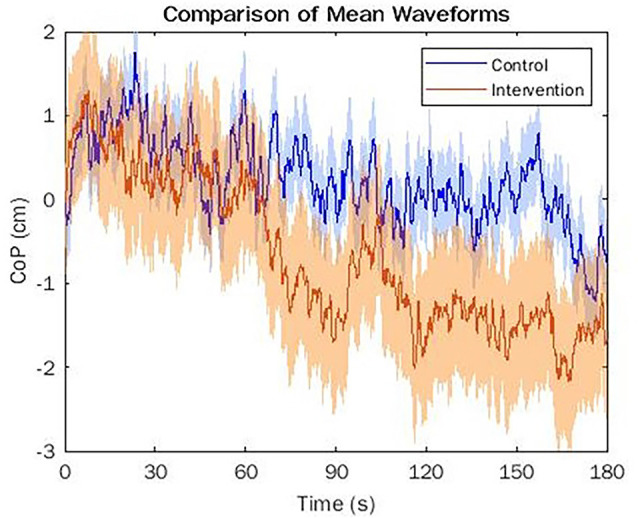
This figure details the mean waveforms of both the control (blue) and the intervention (orange) conditions for the center of pressure (CoP) position with two standard deviation range highlighted for each (shaded blue and shaded orange, respectively). Zero indicates the initial position. Negative values denote a CoP position posterior to initial position and positive values indicate a CoP position anterior to the initial position.

A comparison of mean CoP position waveforms suggests that both groups initially exhibited an anterior sway compared to initial CoP positioning due to the incline illusion (see Figure 4). The control group displayed a slow posterior drift towards the initial CoP position after about 30 s from the initial recording, increasing in magnitude at 60 s and moving posteriorly beyond the initial position at about 120 s (see Figure 4). The intervention group displayed posterior motion earlier than the control group, after about 15 s from the initial recording, and exhibited a larger magnitude posterior shift comparatively. This group moved posteriorly beyond the initial position after about 30 s and remained almost 2 cm posterior for the remaining 2.5 min of the trial.

## Discussion

This study investigated the influence of joint manipulation of the lower-extremities on the development and presence of a centrally mediated, somatosensory-based incline illusion. Bilateral manipulation of the coxofemoral, tibiofemoral, and talotibial joints appears to alter CoP position during an incline illusion compared to a control condition. These differences in CoP (based on CoP time series data) appeared at two intervals in the middle (70–86 s) and late-stage (141–178 s) of a 3-min illusion de-adaptation period. Further, participants in the manipulation group moved less overall (as measured by pathlength) during the final period. They also exhibited lower amplitude movements during the final period, though this was not significant after a correction for multiple comparisons.

Previous research has found changes in movement times (Smith et al., 2006), joint position sense (Haavik and Murphy, 2011), and range of motion (Silva et al., 2019) after spinal manipulation. Despite the local application of stimulus in spinal manipulation (i.e., the spine), larger behavioral and neurophysiological effects have been seen. This has led to postulations that these non-local changes are driven by downstream cortical stimulation, rather than spinal or local influences (Haavik et al., 2016). Studies by Malaya et al. (2020), Malaya et al. (2021) have found similarly non-local effects of upper and lower-extremity manipulation across a variety of tasks; in particular, upper and lower-extremity manipulation appears to influence postural sway, dynamic and standing stability, as well as the overall rhythmicity of movements across both upper and lower-extremity tasking.

This study found that manipulation of the lower-extremities influenced both CoP position and overall movement distance during a centrally mediated somatosensory illusion. Sensory processing mechanisms are thought to be stored as global kinematic coordinates within the dorsospino cerebellar tract with reference specifically to whole-limb coordination as opposed to joint specific references (Bosco and Poppele, 2001). This is the hypothesized mechanism by Kluzik et al. (2007b) for the observed trunk and head lean while the legs were blocked in their post-incline illusion. An afferent barrage of somatosensory information *via* Ia afferents could explain how extremity manipulation alters centrally adapted postural control (Taylor and Murphy, 2008).

Previous studies by Pickar (2002) and Pickar and Bolton (2012) have suggested that spinal and extremity joint manipulations influence perceptual attenuation of joint position sense, leading to greater afferentation from peripheral receptors. The findings of this study support this line of thinking in that alterations in CoP during a centrally-mediated illusion were moderated by peripherally-based joint manipulations. It is possible these effects could point towards alterations in whole-limb reference frames in global kinematic coordinates within the dorsospino cerebellar tract; however, that is beyond the scope of this study. This study contributes to the growing evidence that manipulation of the extremities drives non-local changes in behavior and furthers this concept by suggesting these changes may be driven by alterations in central postural adaptations.

This study furthers our understanding of the influence of extremity manipulation on postural behaviors; however, several important questions remain unanswered. The time course of these postural changes is still unknown. While this study suggests the influence of extremity manipulation can extend to at least 2.5 min, concerted work is needed to fully understand the temporal extent of manipulative interventions. Further, the ability of extremity manipulation to influence CoP position implicates it as a potential therapy for clinical populations in which CoP instability and falls are a major health risk, such as the elderly (Daley and Spinks, 2000; Thomas et al., 2019).

There are several limitations to this study that impact the interpretation of these results. First, given the large standard deviations across CoP measures (and previous work utilizing incline illusions, see Kluzik et al., 2007a), it is likely that the participants of this study could be separated into “responders” and “non-responders” to the incline illusion, and quite possibly, to manipulation in general. To be more specific, it is possible that some participants were influenced by the incline illusion, and some were not. Similarly, some participants may have been influenced by manipulation, and some may not have been. However, this study was only interested in eliciting the presence or absence of a group effect, rather than individual variations to response; future work has been planned to specifically investigate this. This pilot study also has a small sample size. While the significant findings above suggest that the effects of lower-extremity manipulation are robust enough to elicit a significant group effect, a reproduction of this study should recruit more participants and examine individual responses and variation, in addition to group effects. Our authors decided against physical touching or sham manipulation in order to minimize any additional changes to peripheral muscle spindles outside of the development of the incline illusion. No consensus has been made on the gold-standard control or “sham” manipulation in the relevant literature. Future studies may address the effect of physically touching the hip/knee/foot as a sham/control manipulation. That being said, as several functional imaging studies have established central mechanisms for postural control (Duclos et al., 2007; Wright, 2011), the present authors felt that establishing extremity-manipulation’s effect on an established centrally-mediated postural illusion was an acceptable starting place for further research into this area. Future studies should explore the use of EEG during the development of the incline illusion and post-incline postural adaptation to gain a better understanding of the specific effect manipulation has on centrally-mediated postural adaptations as well as other central processing mechanisms.

## Conclusion

Participants in this study were adapted to a centrally mediated somatosensory illusion. After receiving bilateral lower-extremity manipulations, participants exhibited significantly different center of pressure positions and decreased pathlength as compared to controls. The results of this study show that extremity-manipulation alters a centrally-mediated postural illusion, and adds to the body of evidence suggesting that extremity-manipulation has central effects similar to that of spinal manipulation. Further, extremity manipulation may be a useful intervention in clinical populations where the center of pressure instability can lead to falls and injury.

## Data Availability Statement

The raw data supporting the conclusions of this article will be made available by the authors, without undue reservation.

## Ethics Statement

The studies involving human participants were reviewed and approved by Parker University Institutional Review Board. The patients/participants provided their written informed consent to participate in this study.

## Author Contributions

SS designed the research, collected data, interpreted the results, drafted and revised the manuscript. HH and HP participated in designing the research and collecting data. CM designed the research, collected data, interpreted results, performed statistical analysis, drafted and revised the manuscript. DS and CL revised the manuscript. All authors contributed to the article and approved the submitted version.
